# Prediction for 2-year mortality of metastatic ovarian cancer patients based on surveillance, epidemiology, and end results database

**DOI:** 10.3389/fsurg.2022.974536

**Published:** 2022-09-15

**Authors:** Yongxin Wang, Xue Shan, He Dong, Man Li, Ying Yue

**Affiliations:** ^1^ Department of Gynecologic Oncology, the First Hospital of Jilin University, Changchun, China; ^2^ Department of Cardiac Surgery, the First Hospital of Jilin University, Changchun, China

**Keywords:** ovarian cancer, metastasis, prediction, two-year mortality, SEER

## Abstract

**Aim:**

To establish prediction models for 2-year overall survival of ovarian cancer patients with metastasis.

**Methods:**

In total, 4,929 participants from Surveillance, Epidemiology, and End Results (SEER) database were randomly divided into the training set (*n* = 3,451) and the testing set (*n* = 1,478). Univariate and multivariable regression were conducted in the training set to identify predictors for 2-year overall survival of metastatic ovarian cancer patients. The C-index was calculated for assessing the performance of the models. The nomogram for the model was plotted. The prediction value of the model was validated in the testing set. Subgroup analysis were performed concerning surgery and chemotherapy status of patients and the metastatic site of ovarian cancer in the testing set. The calibration curves were plotted and the decision curve analysis (DCA) were conducted.

**Results:**

At the end of follow-up, 2,587 patients were survived and 2,342 patients were dead within 2 years. The 2-year survival rate was 52.5%. The prediction models were constructed based on predictors including age, radiation, surgery and chemotherapy, CA125, and bone, liver, and lung metastasis. The prediction model for 2-year overall survival of ovarian cancer patients with metastasis showed good predictive ability with the C-index of the model of 0.719 (95% CI: 0.706–0.731) in the training set and 0.718 (95% CI: 0.698–0.737) in the testing set. In terms of patients with bone metastasis, the C-index was 0.740 (95% CI: 0.652–0.828) for predicting the 2-year overall survival of ovarian cancer patients. The C-index was 0.836 (95% CI: 0.694–0.979) in patients with brain metastasis, 0.755 (95% CI: 0.721–0.788) in patients with liver metastasis and 0.725 (95% CI: 0.686–0.764) in those with lung metastasis for predicting the 2-year overall survival of ovarian cancer patients.

**Conclusion:**

The models showed good predictive performance for 2-year overall survival of metastatic ovarian cancer patients.

## Introduction

Ovarian cancer is a prevalent and deadly malignant cancer in females that is often diagnosed at an advanced stage with extensive metastasis (1). Based on the data of Globocan, there were 295,414 newly diagnosed ovarian cancer patients and 184,799 deaths registered in 2018 (2). A previous study has reported that in the United States, there was about 21,750 newly diagnosed ovarian cancer patients and 13,940 deaths caused by ovarian cancer in 2020 (1). There was 55,342 newly diagnosed ovarian cancer patients in China in 2020, and the incidence was about 2.6% (3). Another study found that about 490,142 deaths due to ovarian was reported among 19,296,319,576 person-years at risk for women in China from 1990 to 2019 (4). Ovarian cancer is often silently spilled and patients were mainly diagnosed as an advanced disease, which becomes one of the most lethal gynecological malignancy (5). Approximately 70% of ovarian cancer patients were diagnosed with synchronous distant metastases due to the limited early, specific symptoms and effective screening strategies (6). Liver was identified to be the most common distant metastatic organ of ovarian cancer in stage IV which accounted for 37%–57%, followed by distant lymph nodes, lung, bone and brain (7, 8). Metastases is a major cause of mortality in ovarian cancer patients, and the 5-year survival rate was only 29% among women diagnosed with distant-metastatic ovarian cancer (9). Despite the continuous improvement of chemotherapy drugs and advancement of surgical techniques, the 5-year survival rate of ovarian cancer patients is still not more than 50% (10). The 2-year recurrence free survival of ovarian cancer patients was about 48%, less than 50% (11). To identify factors associated with the mortality of patients with metastatic ovarian cancer is essential for improving the prognosis of these patients.

The diagnosis at stage IV at the beginning of treatment sorrowfully decreases the confidence of doctors, patients and their families (12). This may affect the selection of treatments in stage IV ovarian cancer patients, especially in less developed regions. Many patients give up treatment in despair, some patients only choose short-term chemotherapy, and some patients choose standard surgery and chemotherapy, and the prognoses were not the same (13–15). The poor prognosis of metastatic ovarian cancer patients may due to the differences of patients’ metastatic sites and conditions (7). On the other hand, it depends on the decision-making of patients and their families for choosing what kind of treatments. At this point, it is particularly important for patients to make decisions about which treatment can achieve the maximum benefit (16). So the prediction model for the prognosis of metastatic ovarian cancer patients is necessary for both the clinicians and patients choosing the best treatment despite their stages.

In previous studies, various prediction models were established to evaluate the survival of ovarian cancer patients. Zhao et al. constructed a prediction model and plotted a nomogram for predicting the cancer-specific survival of stage II–IV epithelial ovarian cancer patients receiving surgery and chemotherapy (17). Inci et al. conducted a prospective study and established a prediction model for the surgical outcome in ovarian cancer based on frailty index (18). Wang et al. built a prediction model for the survival outcome among epithelial ovarian cancer patients with site-distant metastases (19). Up to date, the prediction model for the survival of all ovarian cancer population such as non-epithelial ovarian cancer patients with metastasis was still lacking (20).

In this study, based on the data from Surveillance, Epidemiology, and End Results (SEER) database, we planned to establish prediction models to predict the 2-year overall survival of metastatic ovarian cancer patients for identifying those who at high possibility to survive within 2 years. Subgroup analysis was conducted concerning the surgery status of patients and the metastatic site of ovarian cancer.

## Methods

### Study design and population

The current study was a cohort study collecting the data of 110,579 ovarian cancer patients from SEER database between 2010 and 2015. SEER database (seer.cancer.gov) is a free access database collecting demographics, tumor characteristics, nodal staging, surgery information, vital status, and follow-up information of patients from 18 cancer registries, which covers about 27.8% of the U.S. population (21). Ovarian cancer in patients was defined by the Site recode ICD-0-3/WHO 2008 (International Classification of Diseases for Oncology, 3rd edition). Patients who were diagnosed with ovarian cancer at the age <18 years old or >80 years old were excluded. Patients without metastasis (M0 stage) were not analyzed. Those who lost the data on surgery, cancer antigen 125 (CA125) and follow-up were excluded. Subjects who were unclear about whether they received surgery were also excluded. Finally, 4,929 subjects were involved and followed up in our study. At the end of the follow-up, 2,587 patients were survived and 2,342 patients were dead.

### Potential prognostic predictors

The potential predictors for the 2-year survival of metastatic ovarian cancer patients were collected including age at diagnosis (years), race (American, Asian, Black, White or unknown), marital status (married, separated, divorced, single, widowed, unknown or unmarried), tumor size (0–1, 1–5 or 5–10 cm), Grade (I, II, III, IV or unknown), AJCC T stage (T0, T1, T2, T3 or unknown), AJCC N stage (N0, N1 or unknown), laterality (one side or paired site), receiving radiation treatment (none/unknown, refused or yes), receiving chemotherapy treatment (no/unknown or yes), surgery (satisfied, unsatisfied or without surgery), receiving surgery and chemotherapy (unsatisfied surgery and post-operative chemotherapy, unsatisfied surgery and pre-operative chemotherapy, unsatisfied surgery and no/unknown chemotherapy, satisfied surgery and post-operative chemotherapy, satisfied surgery and pre-operative chemotherapy, satisfied surgery and no/unknown chemotherapy, without surgery but with chemotherapy or without surgery and no/unknown chemotherapy), CA125 positive (>35 U/ml) or not, bone, brain, liver, and lung metastasis status (no, yes or unknown).

### Outcome variables

The outcome in this study was the overall survival of patients with metastatic ovarian cancer within 2 years. The follow-up time was 2 years and ended in 2015.

### Statistical analysis

The measurement data of normal distribution were shown as mean ± standard deviation (mean ± SD), and the independent sample *t* test was used for comparison between groups. The non-normal data were exhibited as the median quartile [M (Q_1_, Q_3_)], and differences between groups were compared by the Mann-Whitney U rank sum test. Enumeration data were described as *n* (%). Comparison between groups was conducted by chi-square test or Fisher's exact probability method. Statistical analyses were subjected to two-sided test, and the test level was set as *α *= 0.05. All data were randomly sampled. After setting the random seed, 70% of the randomly sampled data were grouped into the training set (*n* = 3,451) and the remaining 30% were used as the testing set (*n* = 1,478). The Kaplan-Meier method was employed for estimating 2-year survival of metastatic ovarian cancer patients in the training set. Then the equilibrium between the training set and testing set was assessed. This study was a cohort study and involved follow-up, Cox proportional hazards model was used for analysis. Univariate and multivariable Cox proportional hazards analyses were conducted in the training set to identify predictors for 2-year overall survival of metastatic ovarian cancer patients. The C-index was calculated for assessing the performance of the models. The nomogram of the model for quickly evaluating the overall survival rate of patients was plotted. The area under the curve (AUC) values of the models for predicting the survival of metastatic ovarian cancer patients at different time were measured. The calibration curves and the decision curve analysis (DCA) were plotted in the training set to evaluate the clinical applicability of the nomogram. Subgroup analysis were performed concerning surgery status of patients and the metastatic site of ovarian cancer in the testing set. SAS 9.4 was applied for data analysis and R 4.0.3 was utilized to establish the prediction model. *P *< 0.05 was regarded as statistical difference.

## Results

### The baseline characteristics of subjects

**Figure 1:**
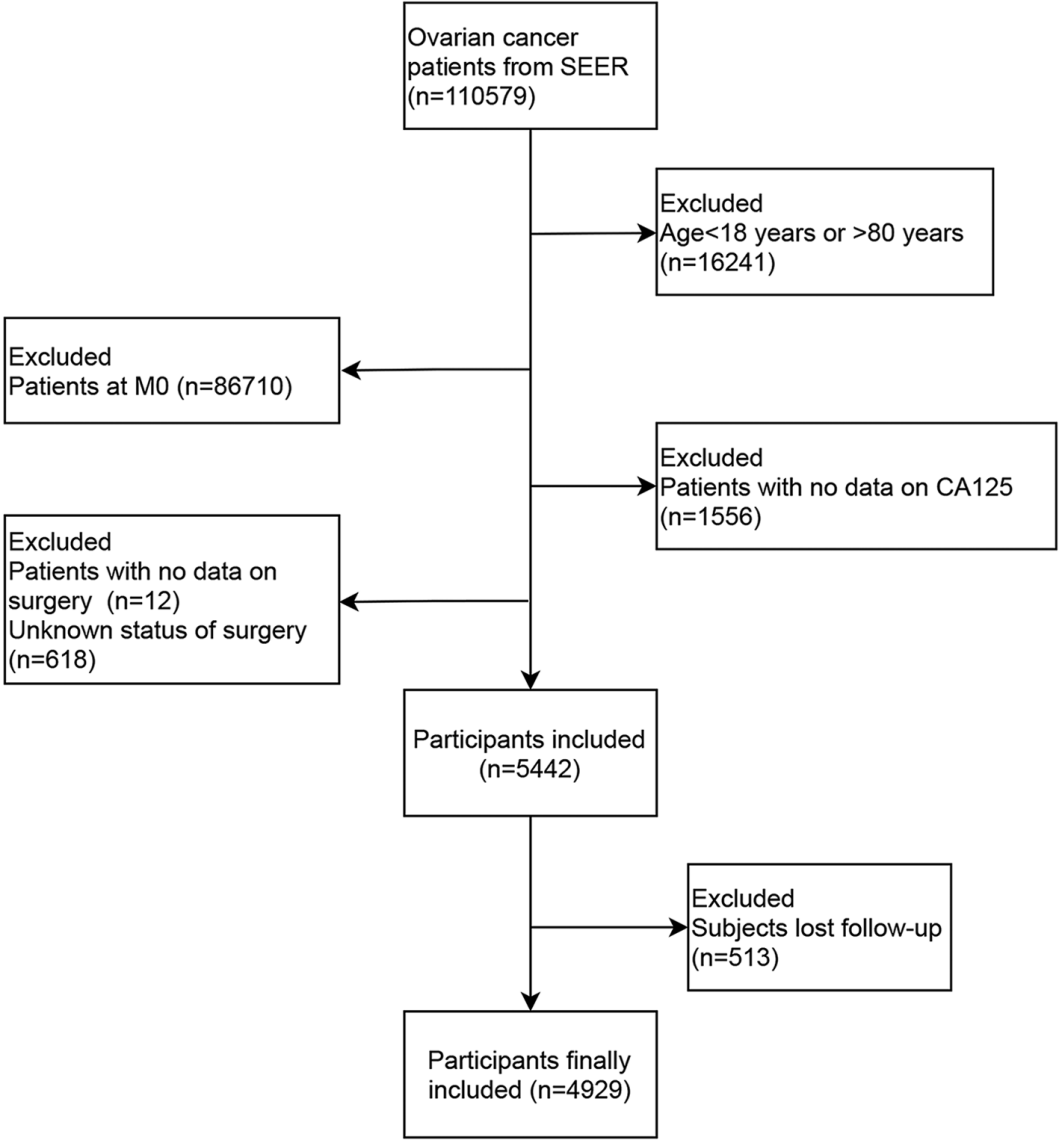
The screen process of the participants in this study.

In the present study, the data of 110,579 ovarian cancer patients from SEER database between 2010 and 2015 were collected. After excluding patients who were diagnosed with ovarian cancer at the age <18 years old or >80 years old (*n* = 16,241), patients at M0 stage (*n* = 86,710), patients who lost the data on surgery (*n* = 12), CA125 (*n* = 1,556) or who were unclear about whether they received surgery or not (*n* = 681), 5,442 patients were included. Among them, 513 patients lost follow-up, and 4,929 patients were finally analyzed. The screen process was presented in Figure 1.

Among all participants, the average age was 62.50 years. 1,919 patients had satisfied results of the surgery, accounting for 38.93%, and 808 patients had unsatisfied results of surgery, accounting for 16.39%. 4,788 patients had positive CA125, which accounted for 97.14%. 151 patients suffered from bone metastasis, accounting for 3.06%, 35 patients had brain metastasis, accounting for 0.71%, 1,258 patients had liver metastasis, accounting for 25.52% and 1,054 patients had lung metastasis, accounting for 21.38%. The median follow-up time was 24 months, and 2,587 patients were survived and 2,342 patients were dead within 2 years. The 2-year survival rate was 52.5% (Table 1).

**Table 1:** Comparisons of the characteristics of patients in the survival group and death group.

Variables	Total (*n* = 4,929)	Group		Statistics	*P*
		Survival group (*n* = 2,587)	Death group (*n* = 2,342)		
Age, mean ± SD	62.50 ± 11.13	61.15 ± 10.97	63.99 ± 11.13	*t *= −9.02	<0.001
Race, *n* (%)				*χ*^2 ^= 35.916	<0.001
American	34 (0.69)	19 (0.73)	15 (0.64)		
Asian	373 (7.57)	219 (8.47)	154 (6.58)		
Black	538 (10.91)	221 (8.54)	317 (13.54)		
Unknown	6 (0.12)	4 (0.15)	2 (0.09)		
White	3,978 (80.71)	2,124 (82.10)	1,854 (79.16)		
Marital status, *n* (%)				*χ*^2 ^= 67.441	<0.001
Divorced	597 (12.11)	302 (11.67)	295 (12.60)		
Married	2,430 (49.30)	1,398 (54.04)	1,032 (44.06)		
Separated	59 (1.20)	33 (1.28)	26 (1.11)		
Single	967 (19.62)	483 (18.67)	484 (20.67)		
Unknown	188 (3.81)	89 (3.44)	99 (4.23)		
Unmarried	16 (0.32)	8 (0.31)	8 (0.34)		
Widowed	672 (13.63)	274 (10.59)	398 (16.99)		
Grade, *n* (%)				*χ*^2 ^= 261.355	<0.001
I	45 (0.91)	33 (1.28)	12 (0.51)		
II	197 (4.00)	130 (5.03)	67 (2.86)		
III	1,359 (27.57)	813 (31.43)	546 (23.31)		
IV	1,079 (21.89)	707 (27.33)	372 (15.88)		
Unknown	2,249 (45.63)	904 (34.94)	1,345 (57.43)		
Tumor size, *n* (%)				*χ*^2 ^= 77.147	<0.001
0–1	1,821 (36.94)	1,061 (41.01)	760 (32.45)		
1–5	1,081 (21.93)	613 (23.70)	468 (19.98)		
5–10	2,027 (41.12)	913 (35.29)	1,114 (47.57)		
Laterality, *n* (%)				*χ*^2 ^= 0.909	0.340
Paired site	3,201 (64.94)	1,696 (65.56)	1,505 (64.26)		
One side	1,728 (35.06)	891 (34.44)	837 (35.74)		
T stage, *n* (%)				*χ*^2 ^= 107.753	<0.001
T1	282 (5.72)	145 (5.60)	137 (5.85)		
T2	435 (8.83)	212 (8.19)	223 (9.52)		
T3	3,545 (71.92)	1,998 (77.23)	1,547 (66.05)		
Unknown	667 (13.53)	232 (8.97)	435 (18.57)		
N stage, *n* (%)				*χ*^2 ^= 57.528	<0.001
N0	2,533 (51.39)	1,365 (52.76)	1,168 (49.87)		
N1	1,662 (33.72)	930 (35.95)	732 (31.26)		
Unknown	734 (14.89)	292 (11.29)	442 (18.87)		
Surgery, *n* (%)				*χ*^2 ^= 556.111	<0.001
Unsatisfied	808 (16.39)	474 (18.32)	334 (14.26)		
Satisfied	1,919 (38.93)	1,356 (52.42)	563 (24.04)		
No	2,202 (44.67)	757 (29.26)	1,445 (61.70)		
Chemotherapy, *n* (%)				*χ*^2 ^= 407.027	<0.001
No/unknown	764 (15.50)	145 (5.60)	619 (26.43)		
Yes	4,165 (84.50)	2,442 (94.40)	1,723 (73.57)		
Radiation, *n* (%)				*χ*^2 ^= 23.138	<0.001
None/unknown	4,818 (97.75)	2,552 (98.65)	2,266 (96.75)		
Refused	17 (0.34)	2 (0.08)	15 (0.64)		
Yes	94 (1.91)	33 (1.28)	61 (2.60)		
Surgery and chemotherapy *n* (%)				*χ*^2 ^= 794.780	<0.001
Unsatisfied surgery and post-operative chemotherapy	580 (11.77)	371 (14.34)	209 (8.92)		
Unsatisfied surgery and pre-operative chemotherapy	144 (2.92)	84 (3.25)	60 (2.56)		
Unsatisfied surgery and no/unknown chemotherapy	84 (1.70)	19 (0.73)	65 (2.78)		
Satisfied surgery and post-operative chemotherapy	1,386 (28.12)	1,020 (39.43)	366 (15.63)		
Satisfied surgery and pre-operative chemotherapy	401 (8.14)	277 (10.71)	124 (5.29)		
Satisfied surgery and no/unknown chemotherapy	132 (2.68)	59 (2.28)	73 (3.12)		
Without surgery but with chemotherapy	1,659 (33.66)	693 (26.79)	966 (41.25)		
Without surgery and no/unknown chemotherapy	543 (11.02)	64 (2.47)	479 (20.45)		
CA125, *n* (%)				*χ*^2 ^= 4.944	0.026
Negative	141 (2.86)	87 (3.36)	54 (2.31)		
Positive	4,788 (97.14)	2,500 (96.64)	2,288 (97.69)		
Bone metastasis, *n* (%)				*χ*^2 ^= 62.129	<0.001
No	4,598 (93.28)	2,475 (95.67)	2,123 (90.65)		
Unknown	180 (3.65)	77 (2.98)	103 (4.40)		
Yes	151 (3.06)	35 (1.35)	116 (4.95)		
Brain metastasis, *n* (%)				*χ*^2 ^= 15.435	<0.001
No	4,701 (95.37)	2,495 (96.44)	2,206 (94.19)		
Unknown	193 (3.92)	81 (3.13)	112 (4.78)		
Yes	35 (0.71)	11 (0.43)	24 (1.02)		
Liver metastasis, *n* (%)				*χ*^2 ^= 35.652	<0.001
No	3,483 (70.66)	1,922 (74.29)	1,561 (66.65)		
Unknown	188 (3.81)	80 (3.09)	108 (4.61)		
Yes	1,258 (25.52)	585 (22.61)	673 (28.74)		
Lung metastasis, *n* (%)				*χ*^2 ^= 29.674	<0.001
No	3,677 (74.60)	2,013 (77.81)	1,664 (71.05)		
Unknown	198 (4.02)	90 (3.48)	108 (4.61)		
Yes	1,054 (21.38)	484 (18.71)	570 (24.34)		
Median survival time	24.00 (8.00, 24.00)	24.00 (24.00, 24.00)	7.00 (2.00, 15.00)	*Z *= −65.674	<0.001

CA125, cancer antigen 125.

### Equilibrium analysis of the data in the training set and the testing set

**Figure 2:**
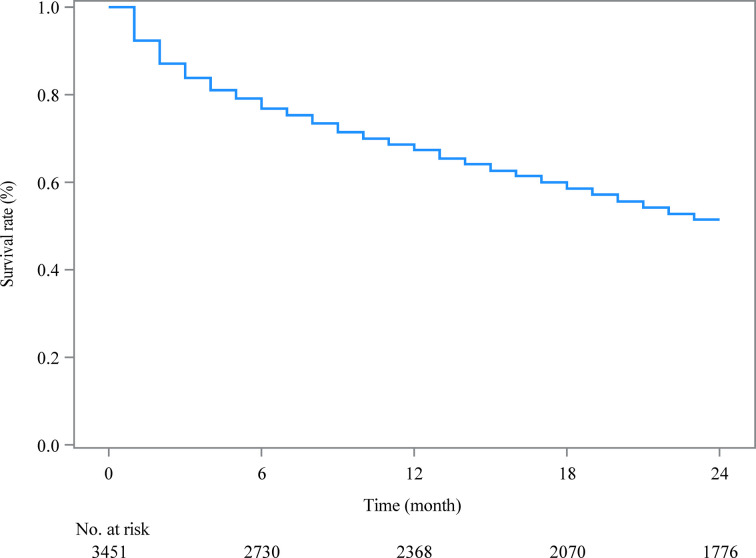
The Kaplan-Meier curve estimating 2-year survival of metastatic ovarian cancer patients in the training set.

As shown in Table 2, after the random division of the training set and the testing set, the results of equilibrium analysis revealed that there was no statistical difference in the demographic and clinical characteristics of patients with metastatic ovarian cancer between the training set and the testing set (all *P *> 0.05). The survival curve of patients in the training set was shown in Figure 2.

**Table 2:** Equilibrium analysis of the data in the training set and the testing set.

Variables	Total (*n* = 4,929)	Group		Statistics	*P*
		Testing set (*n* = 1,478)	Training set (*n* = 3,451)		
Age, mean ± SD	62.50 ± 11.13	62.52 ± 11.29	62.49 ± 11.07	*t* = 0.07	0.945
Race, *n* (%)				*χ*^2 ^= 6.209	0.184
American	34 (0.69)	6 (0.41)	28 (0.81)		
Asian	373 (7.57)	128 (8.66)	245 (7.10)		
Black	538 (10.91)	162 (10.96)	376 (10.90)		
Unknown	6 (0.12)	2 (0.14)	4 (0.12)		
White	3,978 (80.71)	1,180 (79.84)	2,798 (81.08)		
Marital status, *n* (%)				*χ*^2 ^= 1.852	0.933
Divorced	597 (12.11)	182 (12.31)	415 (12.03)		
Married	2,430 (49.30)	738 (49.93)	1,692 (49.03)		
Separated	59 (1.20)	17 (1.15)	42 (1.22)		
Single	967 (19.62)	290 (19.62)	677 (19.62)		
Unknown	188 (3.81)	49 (3.32)	139 (4.03)		
Unmarried	16 (0.32)	5 (0.34)	11 (0.32)		
Widowed	672 (13.63)	197 (13.33)	475 (13.76)		
Grade, *n* (%)				*χ*^2 ^= 1.752	0.781
I	45 (0.91)	17 (1.15)	28 (0.81)		
II	197 (4.00)	55 (3.72)	142 (4.11)		
III	1,359 (27.57)	405 (27.40)	954 (27.64)		
IV	1,079 (21.89)	326 (22.06)	753 (21.82)		
Unknown	2,249 (45.63)	675 (45.67)	1,574 (45.61)		
Tumor size, *n* (%)				*χ*^2 ^= 1.918	0.383
0–1	1,821 (36.94)	525 (35.52)	1,296 (37.55)		
1–5	1,081 (21.93)	328 (22.19)	753 (21.82)		
5–10	2,027 (41.12)	625 (42.29)	1,402 (40.63)		
Laterality, *n* (%)				*χ*^2 ^= 1.352	0.245
Paired site	3,201 (64.94)	942 (63.73)	2,259 (65.46)		
One side	1,728 (35.06)	536 (36.27)	1,192 (34.54)		
T stage, *n* (%)				*χ*^2 ^= 3.998	0.262
T1	282 (5.72)	97 (6.56)	185 (5.36)		
T2	435 (8.83)	120 (8.12)	315 (9.13)		
T3	3,545 (71.92)	1,066 (72.12)	2,479 (71.83)		
Unknown	667 (13.53)	195 (13.19)	472 (13.68)		
N stage, *n* (%)				*χ*^2 ^= 0.260	0.878
N0	2,533 (51.39)	759 (51.35)	1,774 (51.41)		
N1	1,662 (33.72)	504 (34.10)	1,158 (33.56)		
Unknown	734 (14.89)	215 (14.55)	519 (15.04)		
Surgery, *n* (%)				*χ*^2 ^= 1.064	0.587
Unsatisfied	808 (16.39)	230 (15.56)	578 (16.75)		
Satisfied	1,919 (38.93)	581 (39.31)	1,338 (38.77)		
No	2,202 (44.67)	667 (45.13)	1,535 (44.48)		
Chemotherapy, *n* (%)				*χ*^2 ^= 0.483	0.487
No/unknown	764 (15.50)	221 (14.95)	543 (15.73)		
Yes	4,165 (84.50)	1,257 (85.05)	2,908 (84.27)		
Radiation, *n* (%)				*χ*^2 ^= 0.505	0.777
None/unknown	4,818 (97.75)	1,444 (97.70)	3,374 (97.77)		
Refused	17 (0.34)	4 (0.27)	13 (0.38)		
Yes	94 (1.91)	30 (2.03)	64 (1.85)		
Surgery and chemotherapy *n* (%)				*χ*^2 ^= 8.613	0.282
Unsatisfied surgery and post-operative chemotherapy	580 (11.77)	168 (11.37)	412 (11.94)		
Unsatisfied surgery and pre-operative chemotherapy	144 (2.92)	44 (2.98)	100 (2.90)		
Unsatisfied surgery and no/unknown chemotherapy	84 (1.70)	18 (1.22)	66 (1.91)		
Satisfied surgery and post-operative chemotherapy	1,386 (28.12)	403 (27.27)	983 (28.48)		
Satisfied surgery and pre-operative chemotherapy	401 (8.14)	140 (9.47)	261 (7.56)		
Satisfied surgery and no/unknown chemotherapy	132 (2.68)	38 (2.57)	94 (2.72)		
Without surgery but with chemotherapy	1,659 (33.66)	502 (33.96)	1,157 (33.53)		
Without surgery and no/unknown chemotherapy	543 (11.02)	165 (11.16)	378 (10.95)		
CA125, *n* (%)				*χ*^2 ^= 2.073	0.150
Negative	141 (2.86)	50 (3.38)	91 (2.64)		
Positive	4,788 (97.14)	1,428 (96.62)	3,360 (97.36)		
Bone metastasis, *n* (%)				*χ*^2 ^= 1.945	0.378
No	4,598 (93.28)	1,384 (93.64)	3,214 (93.13)		
Unknown	180 (3.65)	46 (3.11)	134 (3.88)		
Yes	151 (3.06)	48 (3.25)	103 (2.98)		
Brain metastasis, *n* (%)				*χ*^2 ^= 5.430	0.066
No	4,701 (95.37)	1,422 (96.21)	3,279 (95.02)		
Unknown	193 (3.92)	51 (3.45)	142 (4.11)		
Yes	35 (0.71)	5 (0.34)	30 (0.87)		
Liver metastasis, *n* (%)				*χ*^2 ^= 2.385	0.303
No	3,483 (70.66)	1,055 (71.38)	2,428 (70.36)		
Unknown	188 (3.81)	47 (3.18)	141 (4.09)		
Yes	1,258 (25.52)	376 (25.44)	882 (25.56)		
Lung metastasis, *n* (%)				*χ*^2 ^= 3.664	0.160
No	3,677 (74.60)	1,094 (74.02)	2,583 (74.85)		
Unknown	198 (4.02)	50 (3.38)	148 (4.29)		
Yes	1,054 (21.38)	334 (22.60)	720 (20.86)		
Survival status, *n* (%)				*χ*^2 ^= 0.041	0.839
Survival	2,587 (52.49)	779 (52.71)	1,808 (52.39)		
Death	2,342 (47.51)	699 (47.29)	1,643 (47.61)		
Follow-up time, M (Q_1_, Q_3_)	24.00 (8.00, 24.00)	24.00 (8.00, 24.00)	24.00 (8.00, 24.00)	*Z *= −0.073	0.942

CA125, cancer antigen 125.

### Predictors for 2-year overall survival of metastatic ovarian cancer patients

According to the data in Table 3, age, race, marital status, grade, tumor size, T stage, N stage, surgery, chemotherapy, radiation therapy, receiving radiation treatment, surgery and chemotherapy treatments, CA125, bone metastasis, brain metastasis, liver metastasis, and lung metastasis were potential predictors for 2-year overall survival of patients with metastatic ovarian cancer.

**Table 3:** Univariate analysis for screening the predictors for 2-year overall survival of metastatic ovarian cancer patients.

Variables	*β*	S.E	*χ* ^2^	Cox	
				HR (95% CI)	*P*
Age	0.018	0.002	56.660	1.02 (1.01–1.02)	<0.001
Race					
American	−0.472	0.309	2.340	0.62 (0.34–1.14)	0.126
Asian	−0.520	0.118	19.466	0.59 (0.47–0.75)	<0.001
Black				Ref	
Unknown	−0.684	1.002	0.465	0.50 (0.07–3.60)	0.495
White	−0.346	0.072	22.910	0.71 (0.61–0.82)	<0.001
Marital status					
Divorced	0.136	0.079	2.924	1.15 (0.98–1.34)	0.087
Married				Ref	
Separated	0.015	0.216	0.005	1.02 (0.66–1.55)	0.944
Single	0.245	0.064	14.495	1.28 (1.13–1.45)	<0.001
Unknown	0.374	0.123	9.155	1.45 (1.14–1.85)	0.002
Unmarried	0.210	0.410	0.262	1.23 (0.55–2.76)	0.608
Widowed	0.451	0.071	40.628	1.57 (1.37–1.80)	<0.001
Grade					
I				Ref	
II	0.019	0.365	0.003	1.02 (0.50–2.08)	0.959
III	0.217	0.337	0.413	1.24 (0.64–2.41)	0.520
IV	0.018	0.339	0.003	1.02 (0.52–1.98)	0.957
Unknown	0.799	0.335	5.686	2.22 (1.15–4.28)	0.017
Tumor size					
0–1				Ref	
1–5	0.121	0.069	3.071	1.13 (0.99–1.29)	0.080
5–10	0.406	0.056	52.953	1.50 (1.35–1.67)	<0.001
Laterality					
Paired site				Ref	
One side	0.046	0.051	0.800	1.05 (0.95–1.16)	0.371
T stage					
T1				Ref	
T2	−0.003	0.130	0.000	0.99 (0.77–1.29)	0.982
T3	−0.193	0.106	3.284	0.82 (0.67–1.02)	0.070
Unknown	0.506	0.116	18.979	1.66 (1.32–2.08)	<0.001
N stage					
N0				Ref	
N1	−0.045	0.056	0.665	0.96 (0.86–1.07)	0.415
Unknown	0.473	0.066	51.541	1.61 (1.41–1.83)	<0.001
Surgery					
Unsatisfied	−0.745	0.071	108.731	0.47 (0.41–0.55)	<0.001
Satisfied	−1.180	0.059	400.801	0.31 (0.27–0.35)	<0.001
No				Ref	
Chemotherapy					
No/Unknown				Ref	
Yes	−1.389	0.057	599.332	0.25 (0.22–0.28)	<0.001
Radiation					
None/Unknown				Ref	
Refused	1.267	0.303	17.518	3.55 (1.96–6.42)	<0.001
Yes	0.506	0.150	11.418	1.66 (1.24–2.22)	<0.001
Surgery and chemotherapy *n* (%)					
Unsatisfied surgery and post-operative chemotherapy	−0.726	0.091	63.060	0.48 (0.40–0.58)	<0.001
Unsatisfied surgery and pre-operative chemotherapy	−0.562	0.155	13.077	0.57 (0.42–0.77)	<0.001
Unsatisfied surgery and no/unknown chemotherapy	0.719	0.141	25.829	2.05 (1.56–2.71)	<0.001
Satisfied surgery and post-operative chemotherapy	−1.066	0.072	217.203	0.34 (0.30–0.40)	<0.001
Satisfied surgery and pre-operative chemotherapy	−0.912	0.112	66.883	0.40 (0.32–0.50)	<0.001
Satisfied surgery and no/unknown chemotherapy	0.069	0.146	0.220	1.07 (0.80–1.43)	0.639
Without surgery but with chemotherapy				Ref	
Without surgery and no/unknown chemotherapy	1.171	0.068	296.914	3.23 (2.82–3.69)	<0.001
CA125					
Negative				Ref	
Positive	0.343	0.166	4.256	1.41 (1.02–1.95)	0.039
Bone metastasis					
No				Ref	
Unknown	0.382	0.114	11.224	1.47 (1.17–1.83)	<0.001
Yes	0.866	0.113	58.786	2.38 (1.91–2.97)	<0.001
Brain metastasis					
No				Ref	
Unknown	0.337	0.111	9.267	1.40 (1.13–1.74)	0.002
Yes	0.948	0.269	12.455	2.58 (1.52–4.37)	<0.001
Liver metastasis					
No				Ref	
Unknown	0.377	0.113	11.210	1.46 (1.17–1.82)	<0.001
Yes	0.299	0.055	29.923	1.35 (1.21–1.50)	<0.001
Lung metastasis					
No				Ref	
Unknown	0.347	0.115	9.151	1.42 (1.13–1.77)	0.002
Yes	0.254	0.057	19.872	1.29 (1.15–1.44)	<0.001

CA125, cancer antigen 125; HR, Hazard ratio; CI, confidence interval.

The results of multivariate cox regression delineated that older ager was associated with higher risk of 2-year overall mortality (HR = 1.01, 95% CI: 1.01–1.01) among metastatic ovarian cancer patients. Patients with unsatisfied surgery and post-operative chemotherapy were linked with decreased risk of 2-year overall mortality (HR = 0.51, 95% CI: 0.43–0.61). Patients received unsatisfied surgery and pre-operative chemotherapy were correlated with decreased risk of 2-year overall mortality (HR = 0.58, 95% CI: 0.43–0.79). The 2.30-fold risk of 2-year overall mortality (HR = 2.30, 95% CI: 1.74–3.04) was observed in patients received unsatisfied surgery and no/unknown chemotherapy. Patients with satisfied surgery and post-operative chemotherapy (HR = 0.37, 95% CI: 0.32–0.42) as well as satisfied surgery and pre-operative chemotherapy (HR = 0.40, 95% CI: 0.32–0.49) were linked with decreased risk of 2-year overall mortality. For those without surgery and no/unknown chemotherapy, the risk of 2-year overall mortality (HR = 3.28, 95% CI: 2.87–3.76) was elevated. The risk of 2-year overall mortality (HR = 1.87, 95% CI: 1.35–2.60) was found in patients with positive CA125. Those with bone (HR = 1.56, 95% CI: 1.25–1.96), liver (HR = 1.34, 95% CI: 1.20–1.49) or lung metastasis (HR = 1.22, 95% CI: 1.09–1.37) were associated with elevated risk of overall mortality (Table 4).

**Table 4:** Multivariable analysis for screening the predictors for 2-year overall survival of metastatic ovarian cancer patients.

Variables	*β*	S.E	*χ* ^2^	Cox	
				HR (95% CI)	*P*
Age	0.009	0.002	14.256	1.01 (1.01–1.01)	<0.001
Radiation					
None/Unknown				Ref	
Refused	0.162	0.307	0.277	1.18 (0.64–2.15)	0.599
Yes	0.476	0.153	9.727	1.61 (1.19–2.17)	0.002
Surgery and chemotherapy					
Unsatisfied surgery and post-operative chemotherapy	−0.673	0.092	53.459	0.51 (0.43–0.61)	<0.001
Unsatisfied surgery and pre-operative chemotherapy	−0.543	0.156	12.158	0.58 (0.43–0.79)	<0.001
Unsatisfied surgery and no/unknown chemotherapy	0.831	0.143	33.973	2.30 (1.74–3.04)	<0.001
Satisfied surgery and post-operative chemotherapy	−0.999	0.073	185.682	0.37 (0.32–0.42)	<0.001
Satisfied surgery and pre-operative chemotherapy	−0.923	0.112	68.069	0.40 (0.32–0.49)	<0.001
Satisfied surgery and no/unknown chemotherapy	0.122	0.148	0.676	1.13 (0.85–1.51)	0.411
Without surgery but with chemotherapy				Ref	
Without surgery and no/unknown chemotherapy	1.188	0.069	294.808	3.28 (2.87–3.76)	<0.001
CA125					
Negative				Ref	
Positive	0.626	0.168	13.947	1.87 (1.35–2.60)	<0.001
Bone metastasis					
No				Ref	
Unknown	0.073	0.181	0.163	1.08 (0.75–1.53)	0.686
Yes	0.447	0.116	14.805	1.56 (1.25–1.96)	<0.001
Liver metastasis					
No				Ref	
Unknown	0.075	0.178	0.179	1.08 (0.76–1.53)	0.672
Yes	0.292	0.055	27.968	1.34 (1.20–1.49)	<0.001
Lung metastasis					
No				Ref	
Unknown	−0.100	0.163	0.376	0.91 (0.66–1.24)	0.540
Yes	0.199	0.059	11.506	1.22 (1.09–1.37)	<0.001

CA125, cancer antigen 125; HR, Hazard ratio; CI, confidence interval.

### Construction and validation of the prediction model for 2-year overall survival of metastatic ovarian cancer patients

**Figure 3:**
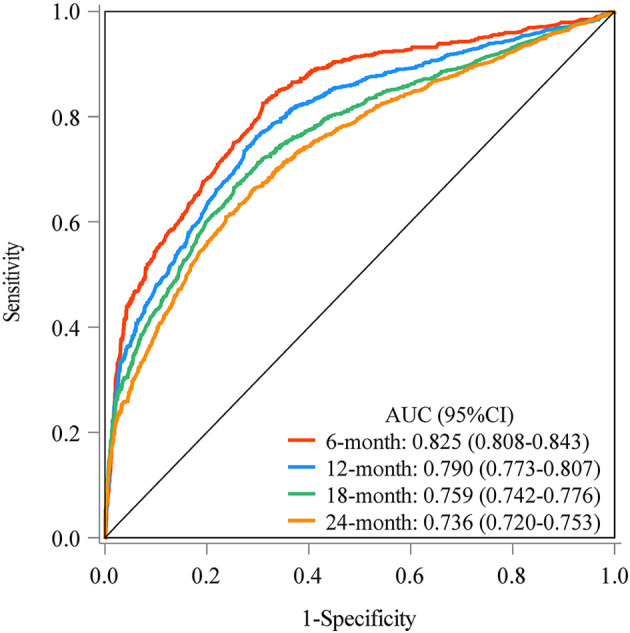
The AUCs of the model for predicting the overall survival of metastatic ovarian cancer patients at different time points in the training set.

**Figure 4:**
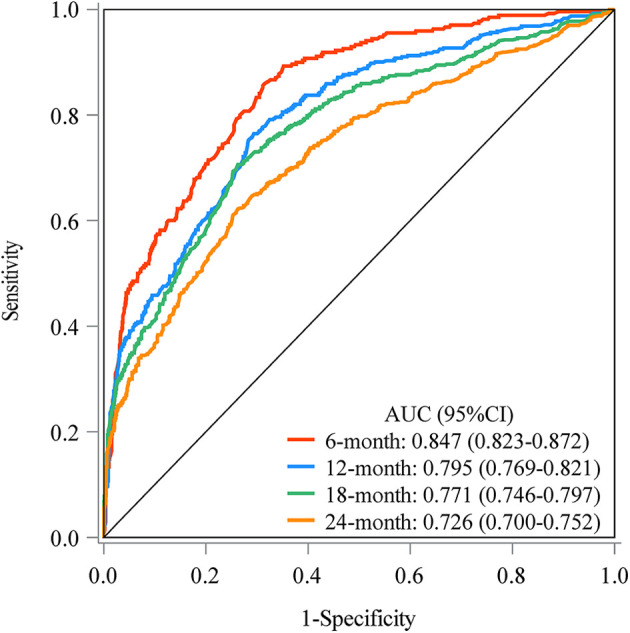
The AUCs of the model for predicting the overall survival of metastatic ovarian cancer patients at different time points in the testing set.

**Figure 5:**
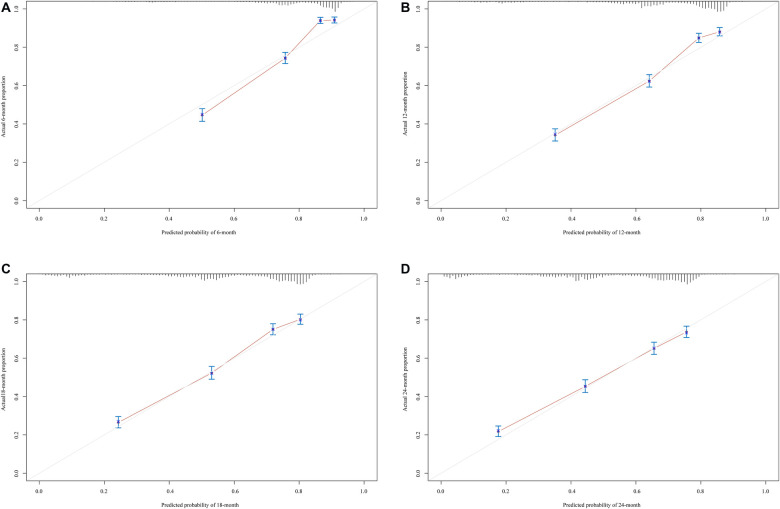
The calibration curves of the prediction model for predicting the 6-month mortality (**A**), 1-year mortality (**B**) and 18-month mortality (**C**) and 2-year mortality (**D**) of metastatic ovarian cancer patients.

**Figure 6:**
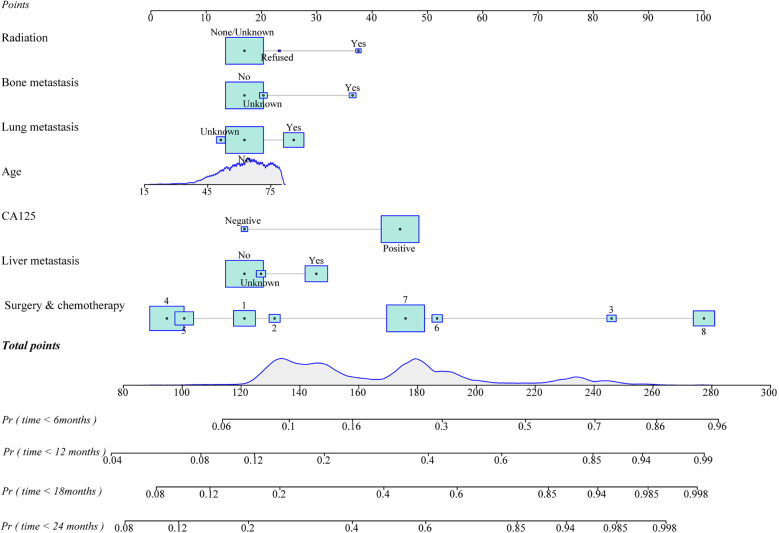
The nomogram of the prediction model for overall survival of metastatic ovarian cancer patients at different time points (1. unsatisfied surgery and post-operative chemotherapy, 2. unsatisfied surgery and pre-operative chemotherapy, 3. unsatisfied surgery and no/unknown chemotherapy, 4. satisfied surgery and post-operative chemotherapy, 5. satisfied surgery and pre-operative chemotherapy, 6. satisfied surgery and no/unknown chemotherapy, 7. without surgery but with chemotherapy or without surgery and 8. no/unknown chemotherapy).

The prediction model was established based on the predictors including age, radiation therapy, surgery and chemotherapy, CA125, bone metastasis, brain metastasis, liver metastasis, and lung metastasis. The C-index of the model for predicting the 2-year overall survival of metastatic ovarian cancer patients was 0.719 (95% CI: 0.706–0.731) in the training set and 0.718 (95% CI: 0.698–0.737) in the testing set (Table 5). The AUCs of the model for predicting the overall survival of metastatic ovarian cancer patients was 0.825 (95% CI: 0.808–0.843) at 6-month, 0.790 (95% CI: 0.773–0.807) at 1-year, 0.759 (95% CI: 0.742–0.776) at 18-month and 0.736 (95% CI: 0.720–0.753) at 2-year in the training set (Figure 3). In the testing set, the AUCs was 0.847 (95% CI: 0.823–0.872) for predicting 6-month overall survival, 0.795 (95% CI: 0.769–0.821) for predicting 1-year overall survival, 0.771 (95% CI: 0.746–0.797) for predicting 18-month survival and 0.726 (95% CI: 0.700–0.752) for predicting 2-year overall survival (Figure 4). The calibration curves of the prediction model for predicting the 6-month mortality (Figure 5A), 1-year mortality (Figure 5B), 18-month mortality (Figure 5C) and 2-year mortality (Figure 5D) of metastatic ovarian cancer patients were shown, which depicted that the prediction values of the model in the training set deviated slightly from the perfected model, but was close to matching, indicating the prediction model had good agreement between the predictive probability and the actual probability. The nomogram for the model was presented in Figure 6. The DCA curve revealed that the use of the nomogram to predict the 2-year overall survival of ovarian cancer patients increased the net benefit than use no model, suggesting that the model might help the clinicians quickly identify those at high risk of 2-year mortality (Supplementary Figure S1).

**Table 5:** The predictive values of the prediction model.

	Training set	Testing set
C-value (95% CI)	0.719 (0.706–0.731)	0.718 (0.698–0.737)
Time depend AUC (95% CI)		
6 months	0.825 (0.808–0.843)	0.847 (0.823–0.872)
12 months	0.790 (0.773–0.807)	0.795 (0.769–0.821)
18 months	0.759 (0.742–0.776)	0.771 (0.746–0.797)
24 months	0.736 (0.720–0.753)	0.726 (0.700–0.752)

CI, confidence interval; AUC, area under the curve.

### Subgroup analysis of the predictive value of the model for the 2-year survival of metastatic ovarian cancer patients

In terms of ovarian cancer patients with bone metastasis, the C-index of the model for predicting the 2-year survival was 0.740 (95% CI: 0.652–0.828). The C-index of the model for predicting the 2-year survival was 0.836 (95% CI: 0.694–0.979) in ovarian cancer patients with brain metastasis. With regard to the status of liver metastasis, the C-index of the model for predicting the 2-year survival was 0.755 (95% CI: 0.721–0.788) for those with liver metastasis. The C-index of the model for the prediction of 2-year survival of metastatic ovarian cancer patients with lung metastasis was 0.725 (95% CI: 0.686–0.764) (Table 6). For patients with satisfied surgery and post-operative chemotherapy, the C-index of the model for predicting the 2-year overall survival of metastatic ovarian cancer patients was 0.501 (95% CI: 0.446–0.557). The C-index was 0.563 (95% CI: 0.464–0.662) for those with satisfied surgery and pre-operative chemotherapy.

**Table 6:** The predictive values for 2-year overall survival in the model in different subgroups.

Subgroup	C-index (95% CI)
Bone metastasis	
Yes	0.740 (0.652–0.828)
No	0.711 (0.690–0.732)
Unknown	0.735 (0.635–0.834)
Brain metastasis	
Yes	0.836 (0.694–0.979)
No	0.714 (0.693–0.734)
Unknown	0.764 (0.679–0.850)
Liver metastasis	
Yes	0.755 (0.721–0.788)
No	0.701 (0.676–0.725)
Unknown	0.742 (0.637–0.847)
Lung metastasis	
Yes	0.725 (0.686–0.764)
No	0.716 (0.692–0.739)
Unknown	0.707 (0.615–0.799)
Surgery and chemotherapy *n* (%)	
Unsatisfied surgery and post-operative chemotherapy	0.514 (0.446–0.582)
Unsatisfied surgery and pre-operative chemotherapy	0.599 (0.472–0.726)
Unsatisfied surgery and no/unknown chemotherapy	0.541 (0.265–0.817)
Satisfied surgery and post-operative chemotherapy	0.501 (0.446–0.557)
Satisfied surgery and pre-operative chemotherapy	0.563 (0.464–0.662)
Satisfied surgery and no/unknown chemotherapy	0.562 (0.420–0.704)
Without surgery but with chemotherapy	0.541 (0.506–0.577)
Without surgery and no/unknown chemotherapy	0.582 (0.523–0.641)

CI, confidence interval.

## Discussion

In this study, the data of 4,929 ovarian cancer patients with metastasis were extracted from SEER database to establish a prediction model for the 2-year survival of those patients based on the screened predictors. The nomogram delineated good predictive value for the 2-year overall survival, with the AUC of 0.726 for 2-year survival in the testing set. Subgroup analysis revealed that the model had good predictive value for patients with bone, brain, liver or lung metastasis. The findings of our study might help identify metastatic ovarian cancer patients with high possibility of survival and provide confidence for the clinicians and patients to receiving proper treatment. For those who predicted with risk of death, timely interventions should be applied for improving their prognosis.

The present study constructed prediction models for 2-year mortality of metastatic ovarian cancer patients based on the predictors including age, radiation therapy, surgery and chemotherapy, CA125, bone metastasis, brain metastasis, liver metastasis, and lung metastasis. Currently, several models were established for predicting the cancer-specific survival of stage II–IV epithelial ovarian cancer patients receiving surgery and chemotherapy (17) or for the surgical outcome in ovarian cancer (18) as well as for the survival outcome in epithelial ovarian cancer patients with site-distant metastases (19). There was still no prediction model for the survival of all ovarian cancer population with different types of metastasis. This study constructed a prediction model for 2-year overall survival of ovarian cancer patients with different kinds of metastasis. In our prediction model, the C-index was 0.722 in the training set and 0.721 in the testing set for predicting the 2-year overall survival of metastatic ovarian cancer patients, which suggested that the model had good predictive ability. Additionally, the AUCs and calibration curves of the model at different time point also indicated that the predictive value of the model for 6-month, 1-year, 18-month and 2-year mortality of metastatic ovarian cancer patients were good. The nomogram of the prediction model was plotted, which could quickly and easily calculate the total score of each patient and obtain the probability of 6-month, 1-year, 18-month and 2-year mortality in the patient. The DCA curves indicated the nomograms were clinically useful. Our model might provide an effective and convenient tool for identifying metastatic ovarian cancer patients who were at high risk of mortality within 2 years and timely interventions should be applied for these patients to improve their prognosis. The model could also help find those with high possibility to survive in 2 years. This might greatly improve the confidence of clinicians and adherence of patients for the treatments. Also, subgroup analysis demonstrated that the model had good predictive performance in patients with bone, brain, liver or lung metastasis, these indicated that the model could be applied in evaluating the risk of mortality in specific metastatic ovarian cancer patients.

CA125 has been widely proposed to be a valuable biomarker in cancer cell growth and survival pathways, tumorigenesis, metastasis and prognosis (22). Previous studies reported that CA125 level is increased in 80% of the patients with epithelial ovarian cancer and 90% of women with epithelial ovarian cancer at advanced stage (23). CA125 has been validated as an important independent indicator correlated with the risk of death of ovarian cancer patients (17). Preoperative serum CA125 level was validated to be a predictor for the extent of cytoreduction in patients with advanced stage epithelial ovarian cancer (24). Rong et al. indicated that early clearance of serum CA125 could predict the platinum response and prognosis in patients with ovarian cancer (25). The findings of these studies gave evidence to the results of our study, which showed that CA125 was an essential predictor for the mortality of ovarian cancer patients with metastasis. Compared with patients have lymphatic metastasis, those with organ metastases showed a lower 5-year overall survival and worse prognosis (26). In the current study, ovarian cancer patients with lung, liver, bone and brain metastases were correlated with the survival of these patients. For patients with lung, liver, bone and brain metastases, attention should be paid at the initial diagnosis and fully evaluation of the condition of patients should be conducted to choose the better plan for the treatment of these patients (27).

For ovarian cancer patients at stage IV, due to spread of disease beyond the ovary, the treatment is more challenging (28). Surgery was regarded as a vital part in the treatment of ovarian cancer patients (29, 30). Complete cytoreductive surgery was reported to be an important modifiable prognostic factor influencing the overall survival time of patients with ovarian cancer (31). As a practice recommended by the National Comprehensive Cancer Center (NCCN), complete cytoreduction to no gross residual disease is a vital factor affecting the progression-free and overall survival of ovarian cancer patients at stage IV (32, 33). Another study also indicated that complete surgical cytoreduction had the best survival benefit in advanced epithelial ovarian cancer patients (34). Balkhy et al. indicated that surgery was the cornerstone of treatment for most stages of non-epithelial ovarian cancers, which might correlate with the prognosis of those patients (35). A retrospective review showed that ovarian cancer patients receiving intraperitoneal chemotherapy were associated with improved 10-year survival of these patients (36). There was evidence indicated that in primary debulking surgery combined with first-line chemotherapy might be the standard treatment for advanced epithelial ovarian cancer patients (37). A multicenter, randomized, phase 3 trial found that secondary cytoreduction followed by chemotherapy might increase the progression-free survival compared with chemotherapy alone in patients with platinum-sensitive relapsed ovarian cancer (38). Other studies were also trying to identify the optimal timing for cytoreduction and hyperthermic intraperitoneal chemotherapy in advanced ovarian cancer patients (39, 40). In this study, regardless of the outcomes of surgery and time of chemotherapy, those received surgery treatment might be associated with lower risk of overall mortality compared with patients who did not undergo surgery treatment. The elevated risk of mortality was observed in patients without both surgery and chemotherapy. These suggested that surgery and chemotherapy should be operated in ovarian cancer patients based on the feasibility conditions of patients to improve the outcome of these patients. In addition, chemotherapy might be associated with some side-effects such as fatigue in patients, and intervention strategies should be proposed to improve the management of these patients during their treatment and in the long term (41). In the past, ovarian cancer was found to be a radiosensitive tumor, and radiation was one of the main therapies for patients with epithelial ovarian cancer, which was applied to manage patients with low residual volumes of disease (42). Nowadays, radiotherapy is replaced by some other therapies in the treatment of ovarian cancer as ovarian cancer is easily to metastasize to other organs, especially in the pelvic and abdominal cavity. Too many organs need irradiation, which may cause more complications and result in poor outcomes in these patients (43). Hreshchyshyn et al. identified that for women with epithelial ovarian cancer at stage I receiving surgery treatment, those who subsequently received radiotherapy had highest recurrence rate (44). Clinicians should evaluate the health status of ovarian cancer patients and whether radiotherapy can be applied for patients should be carefully assessed.

Several limitations existed in the current study. Firstly, all the data were extracted from SEER database, and the participants were mainly White people, whether the prediction model was suitable for Chinese people still needs validation in more studies. Secondly, the variables associated with the risk of mortality in patients with metastatic ovarian cancer were not comprehensive, variables such as the basic demographic characteristic including body mass index and laboratory characteristic including the status of BRCA were not included. BRCA status has been widely tested in ovarian cancer patients in recent years and BRCA positive patients are more sensitive to chemotherapy drugs than negative patients (45, 46), which might be associated with the prognosis of patients. Thirdly, the detailed surgery information such as whether adequate lymphadenectomy was performed could not be extracted from SEER (47). Fourthly, external validation of the results of our study were not performed. Studies with more reliable variables especially surgery information, the status of BRCA and external validation of prediction models were required to validate the findings of this study.

## Conclusion

Our study assessed establish prediction models for 2-year overall survival of ovarian cancer patients with metastasis based on the data of 4,929 metastatic ovarian cancer patients from SEER database. The models showed good predictive performance for 2-year overall survival of ovarian cancer patients with metastasis. In addition, the model presented good predictive value for patients with bone, brain, liver and lung metastasis. The results in this study might help identify metastatic ovarian cancer patients with high possibility of survival and provide a reference for the clinicians and patients to choose a better treatment to improve the prognosis of patients with metastatic ovarian cancer.

## Data Availability

Publicly available datasets were analyzed in this study. This data can be found at: SEER database, https://seer.cancer.gov/.
