# Supplementary figures and images for: Prediction for 2-year mortality of metastatic ovarian cancer patients based on surveillance, epidemiology, and end results database
Source: Front Surg. 2022 Sep 15;9:974536. doi: 10.3389/fsurg.2022.974536 (PMC9632980; doi:10.3389/fsurg.2022.974536)

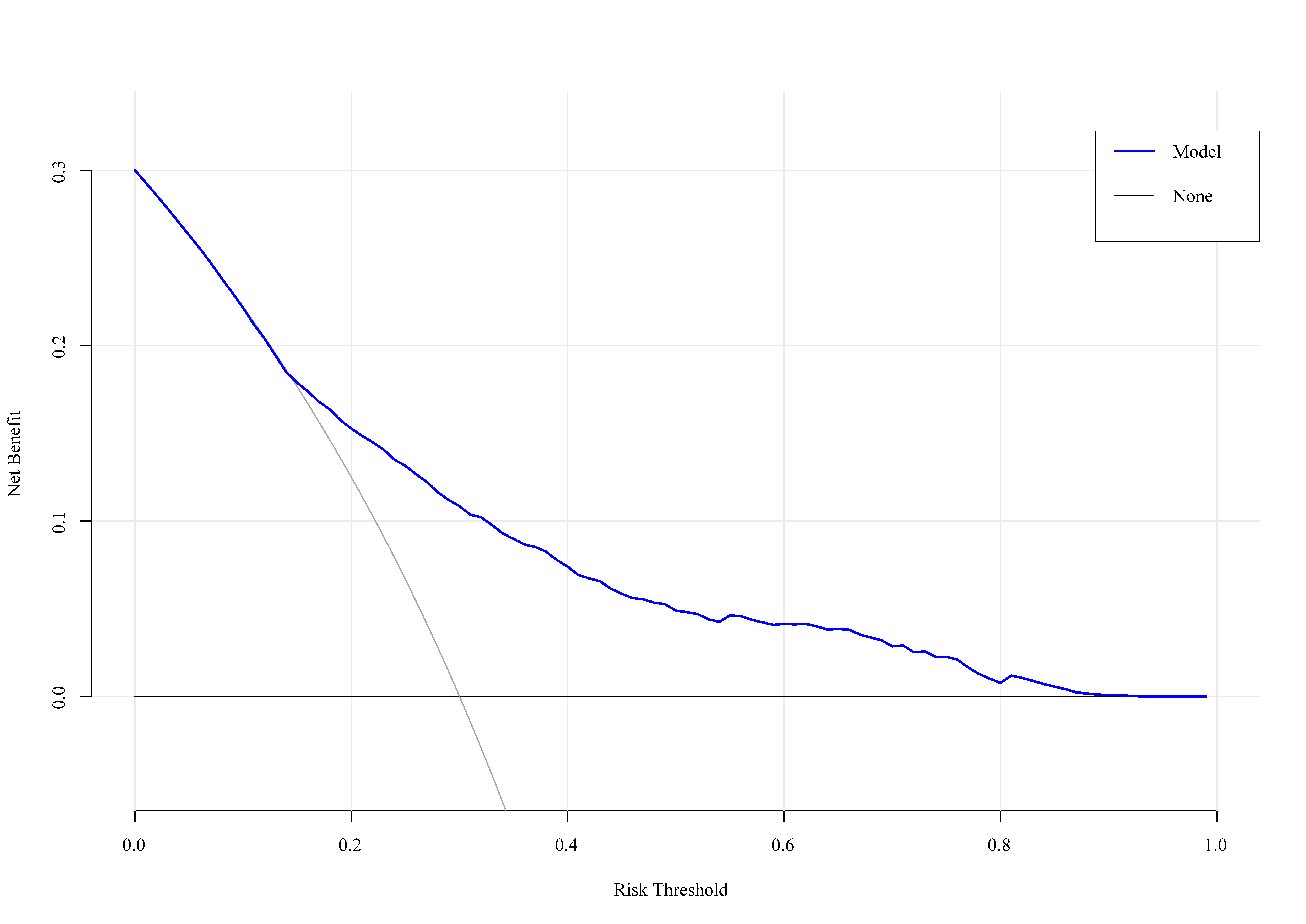

Supplement: Supplementary file 1 [file Image1.tiff]
